# The UK Consensus Statement for the Use of Enzymatic Debridement in Burn Care

**DOI:** 10.3390/ebj7020027

**Published:** 2026-05-12

**Authors:** Nicole Lee, Ascanio Tridente, Niall Martin, Odhran Shelley, on behalf of the UK Working Group for Enzymatic Debridement

**Affiliations:** 1 London and South East Burns Network, London SW10 9NH, UK; niall.martin1@nhs.net; 2 Mersey & West Lancashire NHS Trust, Whiston Hospital, Warrington Road, Prescot L35 5DR, UK; 3 National Burns Unit, St. James’s Hospital, D08 NHY1 Dublin, Ireland; odhran.shelley@nhs.net; 4 Department of Surgery, Trinity College Dublin, D08 NHY1 Dublin, Ireland

**Keywords:** enzymatic debridement

## Abstract

**Background**: Over the past ten years, enzymatic debridement has been used more often to treat burn injuries in the UK and Europe. Even though it is increasingly adopted, there are still major differences in how it is practiced. These differences are mainly due to varying levels of professional experience, differences in the interpretation of available evidence, and concerns about safety and effectiveness. **Methods**: To help resolve these issues and create more consistent care, the UK National Consensus Working Group was formed. This group brought together burn care experts from different backgrounds to review current methods, published research, and consensus guidelines. They used a structured approach that included educational webinars, a thorough review of the literature, and a national survey using the Delphi method to gather expert opinions and real-world experiences. **Results**: As a result of this process, the UK Consensus Statement for the Use of Enzymatic Debridement in Burn Care was created and officially approved after extensive consultation at national meetings. The main recommendations focus on safely and effectively including enzymatic debridement in burn care, the need for clear procedures, and identifying areas where further research is needed, such as patient outcomes and dressing methods. **Conclusions**: The goal of this consensus statement is to unify practices, guide future research, and support ongoing improvements in burn care throughout the UK.

## 1. Introduction

Although enzymatic debridement, including proteases and papain, has been described by Guzman for more than 80 years [1] and used by Meek with good effect [2], it fell out of favour as a treatment. Janžekovič introduced the technique of tangential surgical excision and dermal preservation with excellent outcomes, with her results showing that early return to work brought a new direction of care [3]. Levine and Levenson described the technique and application of bromelain in great detail [4,5], but mainly through animal-based studies; however, Pruitt considered the role of enzymatic therapies and relayed concerns of inadequate debridement, toxicity, debridement of normal tissue and infection [5]. Increased scarring associated with delays in healing noted by Dietch [6], alongside Herendon’s improved outcomes with early total burn excision and wound closure [7], cemented the role for early surgery and grafting. Enzymatic debridement was still used by some and Rosenberg reported the safe, rapid and effective use of bromelain-derived enzymatic agents in a prospective cohort of patients [8]. This resulted in the re-introduction of products using enzymatic agents which are now used in many European and UK burn services today.

Despite numerous studies in the published literature, there is still a paucity of prospective controlled clinical trials, comparing surgery with enzymatic therapies over a long-term follow up. There is also considerable debate regarding the use and variation in enzymatic agents as burn debriding agents in UK practice. These reasons relate to competing strategies, resources and cost. For some clinicians, the use of enzymatic debridement is believed to lead to delays in the definitive management of the burn wound, with some proposing that this treatment modality should be relied upon only in cases where surgery is not an option. The practice of monitoring the wound to assess potential for healing has been deemed responsible for potential increases in treatment costs, without meaningful additional benefit. The perception that enzymatic debridement may not fully align with the concept of early total burn wound excision, considered the cornerstone of modern burn care, and the additional resources needed show that it has not been universally adopted. Those who advocate for enzymatic therapies relate ease of use, enhanced dermal preservation, and good scar outcomes. This has resulted in the coexistence of two distinct strategies in burn care—one of early surgery and grafting, and the other of early application of enzymatic therapies—causing confusion regarding relative indications.

Previously, the European Consensus Guidelines on enzymatic debridement were published by an expert panel of twelve plastic surgeons, burn surgeons, and burn care specialists in 2017 [9] and revised in 2020 [10]. The consensus contained detailed, user-orientated recommendations that attempted to align practice for current and future users while reducing the learning curve that many early adopters had experienced. In early 2021, the ongoing use of enzymatic debridement was discussed at the UK National Burn Operational Delivery Network Morbidity and Mortality Meeting. Several UK burn services started using this technique as part of their management of large burns and experiencing unforeseen challenges, such as cardiovascular instability and bleeding [10]. A need was recognised for improved understanding of the mechanism of action of enzymatic debridement and for harmonising practice across the UK, with the added benefits of facilitating research into relevant clinical outcomes. A UK National Consensus Working Group was convened shortly after to take the process forward.

### Aim

The intention of the UK National Consensus for the Use of Enzymatic Debridement was to standardise the use of the technique and share good practice within the UK.

## 2. Methods

### 2.1. Educational Webinar

Following the initial meeting in early 2021, the working group convened a free online webinar entitled ‘Enzymatic Debridement of Large Burns’, hosted by the National Burns ODN, in November 2021. The webinar was made available to all burn care professionals in the UK, with the intent to explore current practice and identify emerging challenges observed during the treatment of larger burns. The webinar was delivered by experienced users of enzymatic debridement and included an open question-and-answer session as well as feedback from all attendees, which was collated both during the webinar and formally after the event. The participants agreed that clear guidelines were required for the use of enzymatic debridement in the UK. Significant patient safety concerns were raised around the use of enzymatic debridement in large burns, which is an area requiring further evaluation.

### 2.2. Initial Consensus Meeting

Following on from the webinar, they conducted a literature review on the use of enzymatic debridement around the world. Four consensus publications were available at the time with no significant studies being published since the European Consensus Guidelines [9,11,12]. The British Burns Association (BBA) invited interested individuals to join the Working Group to consider a statement on best practice for the UK. Electronic communications to all members of the BBA were sent, with no restriction on who could join the group. Twenty-two healthcare professionals volunteered their time and were invited to an online meeting, held in June 2022, of which 16 were able to attend on the day. All recent published literature and consensus papers were made available to the group before the meeting. The group discussed the use of enzymatic debridement extensively and unanimously agreed that the European Consensus Guidelines was a sufficiently robust and balanced publication that considered the available evidence well. However, the group also agreed that differences in burn care practice across Europe and the UK were sufficiently different that the guidelines themselves could not be adopted directly. The group agreed that the European Consensus Guidelines could be used as the basis for an online survey of current practice in the UK, to gather UK-specific perspectives and ensure that any resulting consensus statement adequately satisfied the national requirement. The online questionnaire was initially piloted by the members of the group, before being disseminated nationally.

### 2.3. Online Survey

Following a literature review of healthcare consensus tools, they concluded that there was strong support for the Delphi approach to data collection with this type of study [13,14]. A survey with 51 questions (47 specific to enzymatic debridement, three demographic questions and one free text) based on the European Consensus Guidelines but containing additional UK-themed questions was devised. The additional questions were based on recommendations from the Working Group and National Burns ODN.

The pilot study was shared with the Working Group in the late summer of 2022. The responses were analysed and minor adjustments were made to the wording of some questions to ensure clarity based on feedback. The agreed scope of the practice was covered sufficiently, and it was recommended that the survey was disseminated to all UK burn care professionals.

The survey was sent to all burn services in the UK and Ireland in autumn 2022 via the BBA, with concurrent emails to all members of the BBA to give everyone a chance to contribute to the process. Although the use of enzymatic debridement has been largely driven by burn surgeons, the supportive role of many other burn care professionals is essential, particularly that of anaesthesic colleagues, to achieve appropriate analgesia and have nursing colleagues to provide wound care. Although individual completion of the survey was implied, there was no restriction on completing the survey as a team. Reminder emails were sent on three occasions, six weeks apart, to ensure that any professional willing to contribute to the survey would be able to do so. The survey was closed after six months, following the third and final email reminder.

### 2.4. Final Consensus Meeting

The survey contained statements to which responders could either agree or disagree. The level of agreement on each statement was graded using the Delphi approach in which consensus is achieved and an intervention or statement is deemed “agreed” if at least 70% of responders are in agreement, or deemed as “strongly agreed” if the proportion is 90% [14,15]. Where consensus could not be achieved, further discussion would be required, requiring an expert panel.

The survey results were presented in the spring of 2023. Forty items (85.1%) achieved outright consensus, leaving seven (14.9%) requiring further discussion. The survey results were presented at the BBA Consensus Meeting, at the international BBA Conference held in Dublin in June 2023.

### 2.5. Review of Consensus Recommendations with Current Literature

Final review of the current literature noted no further change to the statement from UK; this could be due to the removal of product following the change in UK licencing agreements, leaving a period of time that the product was not available. Request calls to publish recommendations with re-introduction of the product in the market in 2025 due to similar challenges were noted in large areas of application.

## 3. Results

The online survey was completed by 47 responders from our 398 members making a 12% uptake on completion of survey. There were some incomplete fields within the dataset, leading to unknown entry if no service or name added data was removed to ensure the quality of data was valid. Two entries were team-based and one duplicated entry was found and removed during data review. This left 44 completed surveys. Responses from 11 burn centres, two units and one facility-level service from all four regions of UK, Scotland, Wales and Ireland were received, with 65% of participants reporting the use of enzymatic debridement at least monthly, 21% quarterly and 14% never used. The responders represented a variety of burn care professionals across the multi-disciplinary team, including 52% surgeons, 2.5% anaesthetists and critical care specialists, as well as 38% nurses, 5% physiotherapists, and 2.5% psychological therapists. Of the 47 clinical questions, 34% received greater than 90% support and were considered to have strong agreement (see Table 1).

An expert panel of 68 burn care professionals with five previous group panel members within this group—leading to 63 new independent members—discussed all aspects of the use of enzymatic debridement at the Final Consensus Meeting and produced a provisional Consensus Statement. This was presented at the BBA Conference and formally ratified as the UK Consensus Statement for the Use of Enzymatic Debridement in Burn Care (see Table 2).

## 4. Discussion

The use of enzymatic debridement in the UK has not been universally adopted and remains controversial in some burn services. considering all the evidence available in the published literature with the opinions and experiences of all burn care professionals who had experience of ongoing or past use of commercially available enzymatic debridement products. It was clear throughout the process that agreement existed for many aspects of the use of enzymatic debridement. Many professionals felt, during discussion, that it had the potential to be a very useful ‘tool in the toolbox’. The initial consensus meeting and online surveys allowed a collaborative approach to build upon the shared learning of others and encourage deeper understanding of what was already known and established and what needed to be further evaluated and understanding. In general, there was agreement with previous European consensus, but with some notable differences. Whereas the European consensus agreed that enzymatic debridement might be less effective in scald injuries, the UK consensus did not agree with this statement. Similarly, the statement that there is not enough evidence to recommend enzymatic debridement for chemical burns was agreed in the European consensus but rejected in the UK consensus. Other differences were a lack of UK consensus of the recommendation to treat certain sites including the face and the perineum. There was also difference in recommendations around timing to grafting following enzymatic debridement and no consensus that this should be delayed for two days. There was broad consensus that patients should be informed of the likely differences in time to heal.

Four key areas were identified for further research in the free text parts of the survey.

- Clear policies and protocols need to be adopted across the UK and elsewhere to ensure that research outcomes are comparable and measure the same end points. The UK Consensus Statement for the Use of Enzymatic Debridement in Burn Care has started this process and it is anticipated that further direction will come from the National Burns ODN and BBA as more evidence becomes available.
- Patient experience is under-represented in the published literature, but it is an important part of the UK National Burn Care Standards. It is anticipated that further direction will come from the National Burns ODN and BBA to encourage all services that use enzymatic debridement to audit their outcomes, including healing times and scarring measures.
- Dressing protocols can have a significant impact on the progression of any remaining dermal elements but can be influenced by numerous external factors, such as organisational and financial factors and constraints. The continued use of allograft for small burns treated with enzymatic debridement is not considered best practice unless there is a clear benefit in terms of the recovery of form and function that outweighs potential risks. Hence, a clearer understanding of which dressings to use following enzymatic debridement is required as a matter of urgency.
- There is an increasing tendency for enzymatic debridement to be used to treat smaller burns in the outpatient day-case settings in some services. This can present challenges in terms of appropriate follow up and senior review by experienced professionals. An educational programme was suggested by a significant number of responders to the survey and from the audience at the BBA Conference to ensure safe and effective burn care is delivered at all times.

The development of a UK Consensus Statement for the Use of Enzymatic Debridement in Burn Care is limited by the availability and heterogeneity of the published evidence, and the somewhat polarised viewpoints on the use of enzymatic debridement that existed in the UK at the time. The statement in Table 1 is, however, sufficiently inclusive to encourage the ongoing use of enzymatic debridement while also highlighting areas for concern, proper planning of resources, and opportunities for future research. Where the evidence is currently lacking, the statement is based on a substantial body of expert opinion and experience in the management of burns, not just the use of enzymatic debridement.

### Limitations

A significant limitation of the consensus statement presented here is the low response rate, at 12% in the present survey. Low response rates are reported as very common in medical surveys, particularly when targeting physicians, with average rates often falling between 30% and 50%, but being as low as 8%, particularly with online surveys [16]. While it may be considered that this weakness could impact the generalisability of the findings, such constraint was unavoidable, due to the nature of the process. Attempts to mitigate for the low response rates were made by implementing multiple waves of email requests and reminders for replies. Future larger studies may be considered in order to overcome these limitations.

## 5. Conclusions

Consensus was achieved in many areas on the use on enzymatic agents, and this paper contains broad recommendations of the safe use of enzymatic debridement based on current evidence and a substantial body of expert opinion. The UK Working Group would like to see the statements refined every five years to have clear indications, well-defined techniques for application, and a better appreciation of long-term outcomes, including scarring and patient experience with increasing datasets to drive practice.

## Figures and Tables

**Table 1:** Online survey results.

Question	Statement	Agree (%)	Agree (*n*)	Disagree (%)	Disagree (*n*)	Answered	Skipped
Q1	Classifications with regard to timing of application are “immediate (or very early) (<12 h)”, early (12–72 h) or delayed (>72 h).	83.72%	36	16.28%	7	43	1
Q2	Enzymatic debridement might be less effective in scald injuries.	37.21%	16	62.79%	27	43	1
Q3	There is not enough evidence to recommend enzymatic debridement for chemical burns.	69.77%	30	30.23%	13	43	1
Q4	Outpatient treatment/enzymatic debridement as a day case can be performed after careful patient selection in minor burns in experienced burn services.	76.74%	33	23.26%	10	43	1
Q5	Repeated application of enzymatic debridement can only be recommended in exceptional cases.	83.72%	36	16.28%	7	43	1
Q6	Enzymatic debridement is best indicated for mid-to deep-dermal burns with mixed patterns.	83.72%	36	16.28%	7	43	1
Q7	Enzymatic debridement can be applied in full-thickness burns.	76.74%	33	25.58%	11	43	1
Q8	Application of enzymatic debridement as early as possible during admission can prevent burn-related compartment syndrome in circumferential extremity burns.	83.33%	35	19.05%	8	42	2
Q9	Enzymatic debridement applied as early as possible during admission might prevent development of burn-induced compartment syndrome in extensive trunk burns.	69.77%	30	30.23%	13	43	1
Q10	Enzymatic debridement cannot replace surgical release for extended trunk burns in case of established respiratory compromise.	93.02%	40	6.98%	3	43	1
Q11	Enzymatic debridement is not recommended for burns of the extremity where there is evidence of established compartment syndrome or a high-voltage injury.	93.02%	40	6.98%	3	43	1
Q12	Enzymatic debridement treatment of burns on the palm or sole might be indicated in selected patients.	86.05%	37	13.95%	6	43	1
Q13	Enzymatic debridement is recommended for facial burns.	58.14%	25	44.19%	19	43	1
Q14	Enzymatic debridement may be used with caution on the face if meticulous protection is afforded to the eyes and orifices.	90.70%	39	9.30%	4	43	1
Q15	Ophthalmological exam after facial burns is recommended prior to and after enzymatic debridement treatment.	88.37%	38	11.63%	5	43	1
Q16	Enzymatic debridement is recommended for perineal and genital burns.	30.23%	13	69.77%	30	43	1
Q17	LDI or FLIR cameras are helpful tools for identification of regions that undergo enzymatic debridement.	60.47%	26	39.53%	17	43	1
Q18	Regional anaesthesia is recommended for enzymatic debridement of the isolated (upper/lower) burnt extremity.	95.24%	40	4.76%	2	42	2
Q19	Local anaesthesia for enzymatic debridement is useful in minor burns.	85.71%	36	14.29%	6	42	2
Q20	Sequential enzymatic debridement is possible for large burns but only if about 10%TBSA is treated at a time (15%TBSA as an absolute maximum).	69.05%	29	33.33%	14	42	2
Q21	Enzymatic debridement of more than 10% TBSA/session requires adequate monitoring, and haemodynamic support is considered as a surgical procedure with anaesthetic support.	100.00%	43	0.00%	0	43	1
Q22	Where sequential enzymatic debridement is proposed, there should be at least 12 h between applications.	74.42%	32	25.58%	11	43	1
Q23	Late application (>72 h from injury) is possible in selected wounds after appropriate prolonged pre-soaking.	72.09%	31	27.91%	12	43	1
Q24	Enzymatic debridement should not be used in the presence of existing coagulopathy.	83.72%	36	16.28%	7	43	1
Q25	Hydrogel dressings can be used as an effective moisturiser for dry eschar to improve pre-soaking.	60.98%	25	39.02%	16	41	3
Q26	Pre-soaking can be scheduled overnight to synchronise the enzymatic debridement application with the day shift team.	95.35%	41	4.65%	2	43	1
Q27	Enzymatic debridement should not be applied to burns that have been covered with silver sulfadiazine or betadine.	90.24%	37	9.76%	4	41	3
Q28	Persistent dry eschar after pre-soaking requires superficial surgical debridement prior to enzymatic debridement.	69.77%	30	30.23%	13	43	1
Q29	Shortening of the application time of the product < 4 h is not recommended.	83.72%	36	16.28%	7	43	1
Q30	Wound bed colour, bleeding patterns and general morphology should be assessed by an experienced burn surgeon/nurse as soon as the enzymatic debridement dressings are removed.	95.35%	41	4.65%	2	43	1
Q31	A management plan with regard to further treatment modalities should be directly defined after enzymatic debridement by an experienced burn surgeon/nurse.	97.67%	42	2.33%	1	43	1
Q32	A second wet soak phase post removal of enzymatic debridement removes further debris from the wound bed and should be performed.	88.37%	38	11.63%	5	43	1
Q33	Membrane dressings and allografts can be applied after wet-to-dry phase in wounds that are expected to heal without autografting.	92.86%	39	7.14%	3	42	2
Q34	Allografts can be applied temporarily in wounds that are not expected to heal spontaneously after enzymatic debridement prior to autografting.	88.10%	37	11.90%	5	42	2
Q35	There is insufficient evidence to recommend an ideal post-enzymatic debridement dressing to reduce the reliance on allograft.	90.70%	39	9.30%	4	43	1
Q36	Indication for administration of antibiotics in the context is equivalent to surgical eschar removal.	59.52%	25	40.48%	17	42	2
Q37	In case of full-thickness burns after enzymatic debridement, autologous skin grafting should be delayed for at least 2 days.	69.77%	30	30.23%	13	43	1
Q38	Deep-dermal burn following enzymatic debridement wounds may benefit from early autografting.	64.29%	27	35.71%	15	42	2
Q39	Autologous skin grafting should be considered after 21 days if there is no significant progress in epithelisation.	81.40%	35	18.60%	8	43	1
Q40	Scar treatment and prevention of hypertrophic scars is performed according to established standard protocols in burn care (compression garments, silicon and abstention from UV radiation).	95.35%	41	4.65%	2	43	1
Q41	Prolonged conservative treatment after enzymatic debridement may result in unstable scarring with intensive wound care, and regular reconsideration should be given for autografting.	80.95%	34	19.05%	8	42	2
Q42	Enzymatic debridement may help to reduce the usage of resources (blood products, surgery, OR room capacity, human resources).	81.40%	35	18.60%	8	43	1
Q43	Data on the patients’ experience on enzymatic debridement are rare and future research on patient experience is needed.	93.18%	41	6.82%	3	44	0
Q44	Patients should be made aware of the difference in time to heal with enzymatic debridement versus surgical excision during consent procedure.	97.67%	42	2.33%	1	43	1

**Table 2:** UK Consensus Statement for the use of enzymatic debridement in burn care.

**Terminology:** The use of enzymatic debridement should be considered as immediate (if applied less than 12 h from the time of burn), early (12–72 h), or delayed (more than 72 h).
**Consent:** Patients must explicitly consent, and fully acknowledge, that the time to heal is likely to be longer compared to surgical excision as this has clear implications for returning to work or normal activities of daily living. The use of allograft including its risks and possible long-term effects must also be discussed.
**General comments:** Enzymatic debridement can be used in mixed pattern mid- to deep-dermal burns. It can also be used to debride full-thickness burns in patients otherwise unfit for early burn wound excision, e.g., complex polytrauma, or where other factors prevent suitable anaesthesia or surgical intervention. The use of enzymatic debridement is probably less effective in scald and corrosive substance burns but more evidence is required.
Enzymatic debridement can be used, with careful patient selection, in an outpatient day-case setting for ‘minor’ burns in experienced burn services. Repeat application of enzymatic debridement to the same anatomical area is not recommended but can be considered in special circumstances on a case-by-case basis.
Enzymatic debridement can be used to prevent burn-related compartment syndrome in circumferential extremity and trunk burns. **Enzymatic debridement must not replace surgical release of torso burns with evidence of established respiratory compromise.** Enzymatic debridement is not recommended for high-risk extremity burns, such as high-voltage electrical injuries or those with established compartment syndrome.
Enzymatic debridement can be used cautiously on facial burns but the eyes and oronasal orifices must be protected meticulously. **When applied to the face, fluorescein eye examination must be performed before and after use.** Enzymatic debridement can also be used on the palms and soles of the feet. Enzymatic debridement is not recommended for perineal and genital burns.
Local anaesthesia can be used for the enzymatic debridement of ‘minor’ burns. If not otherwise contraindicated, regional anaesthesia is recommended for the enzymatic debridement of burns involving more than one anatomical area or those not considered to be ‘minor’.
Enzymatic debridement should only be used within the manufacturer’s guidelines. Sequential enzymatic debridement is possible in ‘major’ burns. Enzymatic debridement of more than 10%TBSA requires haemodynamic monitoring supported by trained healthcare professionals. There should be at least 12 h between sequential applications.
Pre-soaking can be scheduled overnight to synchronise the application of enzymatic debridement within normal working hours. Additional soaking after removal of the enzymatic debridement is recommended. Late enzymatic debridement (i.e., more than 72 h after injury) may be considered in special circumstances on a case-by-case basis after an adequate period of pre-soaking.
The debrided wound must be assessed by appropriately trained healthcare professionals as soon as the product has been removed to confirm a management plan. It is recognised that healthcare professionals may not have sufficient experience when the technique is first introduced. It is recommended that they work within an MDT and consider collaboration with, or mentorship by, more experienced colleagues elsewhere to interpret wounds effectively.
After wound assessment, membrane dressings or allograft can be used where healing is expected without the need for autografting. It is recognised that the aim of any dressing used after enzymatic debridement is to protect dermal elements from desiccation and avoid any progressive necrosis. It is also recognised that burn services should aim to reduce their use of allograft where possible. However, there is currently insufficient evidence to recommend any specific dressing particularly when individual burn services may be logistically or financially constrained by the availability of such dressings.
When enzymatic debridement results in a full-thickness defect, it is recommended that autografting is delayed for 5–7 days. All other wounds must be reviewed regularly by appropriately trained healthcare professionals to determine a satisfactory healing trajectory. It is recommended that any wound with an inadequate healing trajectory at 21 days should be reviewed for excision and autografting. **Prolonged conservative management is not recommended if the wound healing is failing to progress**.
Enzymatic debridement is a specialised service that requires new knowledge and practical skills, adaptation of infrastructure and protocols, and multiprofessional collaboration to ensure that the high standards of burn care in the UK are maintained. It is recognised that the continued use of enzymatic debridement is likely to change the utilisation of resources but there is currently insufficient evidence to understand what impact, beneficial or otherwise, its use will have on individual UK burn services. Further work is required to understand the impact on resources, capabilities, costs, patient experience and outcomes.

## Data Availability

The original contributions presented in this study are included in the article. Further inquiries can be directed to the corresponding author.

## Supplementary Materials

The following supporting information can be downloaded at https://www.mdpi.com/article/10.3390/ebj7020027/s1, Table S1: Working party (the UK Working Group for Enzymatic Debridement) group details.
