# Supplementary material for: The UK Consensus Statement for the Use of Enzymatic Debridement in Burn Care
Source: Eur Burn J. 2026 May 12;7(2):27. doi: 10.3390/ebj7020027 (PMC13214822; doi:10.3390/ebj7020027)
Supplement: Supplementary file 1 [file ebj-07-00027-s001.zip › ebj-4151531-supplementary.pdf]

Working party (the UK Working Group for Enzymatic Debridement) group details

Adorno, Jose	Hospital Santa Lúcia Sul
Alexander, Mr Skaria	Nottingham University NHS Trust
Anwar, Mr Umair	Pinderfields General Hospital
Bates, Kathy	Mid And South Essex Nhs Foundation Trust
Berry, Dr Pete	St Andrew’s Centre, Broomfield
Booth, Mr Simon	University Of Bath
Chipp, Miss Elizabeth	University Hospital Birmingham
Cubitt, Mr Jonathan	The Welsh Centre Of Burns And Plastic Surgery
Drew, Mr Peter	Swansea Burns Centre
Dudman, Mrs Vicky	St Andrews Centre
Fisher, Dr. Mark	Johns Hopkins University
Hemington-gorse, Miss Sarah	Welsh Centre For Burns
Hickerson, Dr. William	UTHSC - Retired
Highway, Mrs Karen	Bristol Adult Burns Unit, Southmead Hospital Bristol North Bristol Nhs Trust
Horigan, Mrs Jessica	Sheffield Children's Hospital
Huddleston, Emily	Birmingham Children's Hospital
Hughes, Dr. William	Thomas Jefferson University Hospital
Ibradzic, Dr Zlatan	St Andrews Burns Center
Imran, Professor Madya Farrah Hani	Universiti Kebangsaan Malaysia (national University Of Malaysia)
Jenkins, Professor Toby	University Of Bath
Kennedy, Miss Mary	Burns Unit, Nottingham University Hospitals NHS Trust
Mccarrick, Una	St James’s Hospital Dublin
Miles, Joanna	Norfolk And Norwich University Hospitals Nhs Foundation Trust
Mohamed, Ms Dalia	The Dudley Group Nhs Foundation Trust
Moortgat, Mr Peter	Oscare
Muthayya, Miss Preetha	Mid Yorkshire Hospitals NHS trust
Nicholls, Mrs liz	sheffield Children's hospital
Philippou, Ms. Katia	Bio Med Sciences
Pontes Andrade, Dr Fernando	Burn Unit - Regional Hospital of North Wing
Prince, Miss Amberley	University Hospitals Birmingham
Seoighe, Dr Deirdre	Waikato Hospital
Shelley, Prof Odhran	St James Hospital Dublin
Sloan, Dr Brendan	Pinderfields Hospital
Spoors, Dr Cath	St Andrew’s Centre For Burns And Plastic Surgery
Stone, Mr Matthew	Hull University Teaching Hospitals
Swales, Mrs Claire	NHS
Tiernan, Mr Eunan	Salisbury District Hospital
Van Wyk, Dr Christopher	MSE Broomfield Hospital Trust
Velamuri, Dr Sai Ramakrishna	University of Tennessee, Memphis, USA
Wearn, Mr Chris	Bristol Adult Burns Service, North Bristol NHS Trust
Wong She, Dr Richard	National Burn Centre Of New Zealand
Yarwood, Dr Jamie	Mid Yorkshire Hospitals Nhs Trust
Arkoulis, Mr Nick	Canniesburn Plastic Surgery Unit
Carter, Dr Andrew	Mid Yorks Nhs Trust
Austin, Miss Orla	Mid Yorkshire Nhs Trust
Bache, Ms Sarah	South Metropolitan Health Service
Bailey, Dr Clare	Chelsea And Westminster Hospital

Barnes, Mr David	St Andrews Burns Service, Broomfield Hospital
Black, Miss Claire	Royal Victoria Hospital
Burge, Mr Tim	Salisbury NHS Foundation Trust
Chung, Christine	Chelsea and Westminster Hospital
Falder, Ms Sian	Alder Hey Children's Nhs Foundation Trust
Fanstone, Dr Ruthann	Tlsc
Hazlehurst, Miss Emma	Sheffield Childrens Hospital
Homen, Ricardo	HRAN - Brasilia
Hughes, Mrs. Michelle	Thomas Jefferson University Hospital
Jalloh, Fatmata	Holy Spirit Hospital
Kloczko, Dr Ewa	University College London Hospital
Lee, Mrs Nicole	Chelsea And Westminster
Leon-Villapalos, Mr Jorge	Chelsea And Westminster Hospital
Mccarroll, Miss Sarah	Sheffield Children’s Hospital
Moazzam, Mr Amber	Middlemore Hospital, Auckland, New Zealand
O'donnell, Ms Michelle	St James's Hospital
Phipps, Mr Alan	Mid Yorkshire Hospitals Nhs Trust
Raraty, Mrs Catherine	Alder Hey Children's Hospital
Rodgers, Mrs Louise	Queen Victoria Hospital
Tariq, Dr Muhammad Hassaan	BURN CARE CENTRE PIMS
Thomas, Miss Clare	Birmingham Children's Hospital
Alazzmi, Miss Hana	Qassim University
